# Early Childhood Educators’ Wellbeing During the COVID-19 Pandemic

**DOI:** 10.1007/s10643-021-01203-3

**Published:** 2021-05-10

**Authors:** Patricia Eadie, Penny Levickis, Lisa Murray, Jane Page, Catriona Elek, Amelia Church

**Affiliations:** 1 Melbourne Graduate School of Education, The University of Melbourne, Level 4, 100 Leicester St, Carlton 3053, Melbourne, Australia; 2 Murdoch Children’s Research Institute, Melbourne, Australia

**Keywords:** Early childhood educators, Wellbeing, COVID-19, Educator-child relationships, Preschool

## Abstract

The importance of Early Childhood (EC) educators’ wellbeing has been brought into sharp focus during the COVID-19 pandemic, as educators have navigated numerous additional stressors while providing education and care services for some children and ongoing support for many others learning at home. This study aimed to explore the impact of the pandemic on EC educators’ wellbeing and educator-child relationships, as growing evidence shows the influence of these factors on children’s developmental outcomes.

In July 2020, members of a Research Network of EC Professionals—who previously identified educator wellbeing as a priority issue—were invited to participate in an online survey. The survey included two published, validated scales: the Early Childhood Professional Wellbeing scale (ECPW) and the Student–Teacher Relationship Scale (modified). Survey items about educators’ experiences during the pandemic were also included. Two hundred and thirty-two EC educators from across Australia completed the survey, mostly from Victoria where lockdowns were most severe. Linear regression analysis demonstrated stronger professional wellbeing was associated with less conflict in educator-child relationships and lower risk of staff turnover. This was more likely to be experienced by senior or more experienced staff. Although a negative impact of COVID-19 was reported, ECPW scores were relatively high, and organizational structures supporting professional wellbeing were most strongly associated with lower risk of turnover (*r* = 0.63, *p* < 0.001). Findings highlight that supporting EC educators’ wellbeing is essential for workforce retention, and for promoting quality educator-child relationships which are central to young children’s learning and development.

## Introduction

There is growing recognition that the early childhood education and care (ECEC) profession faces challenging working conditions, leading to high levels of work-related stress, emotional exhaustion and staff turnover (Irvine et al., 2016; Jena-Crottet, 2017; Jones et al., 2017; McMullen et al., 2020; OECD, 2019; Thorpe et al., 2020; Totenhagen et al., 2016). The role of early childhood (EC) educators is complex and multifaceted, requiring a commitment to continuous improvement, resilience, and a willingness to take on many challenges (Beltman et al., 2019; Irvine et al., 2016; Liu et al., 2018). Recent efforts to raise the quality and professionalism of the workforce has increased demands on EC educators, including requirements for higher qualifications and increased accountability (Cumming et al., 2015). Alongside meeting the increasing demands and expectations of their role, EC educators face numerous challenges including long working hours, low salaries, a lack of status and public recognition, and limited opportunities for professional development or career progression (Cumming et al., 2015; OECD, 2019; Phillips et al., 2016; Thorpe et al., 2020; Whitebook et al., 2014). The consequences of these challenges are reflected in high levels of emotional exhaustion and work-related stress, high staff turnover, and poor EC educator mental health and wellbeing in the early childhood sector (Irvine et al., 2016; Phillips et al., 2016; Totenhagen et al., 2016).

The impact of the COVID-19 pandemic created conditions likely to exacerbate these challenges. In 2020, Australian and especially Victorian EC educators were required to navigate additional stressors in providing ECEC services for highly vulnerable children and children of essential workers, as well as ongoing support for children’s learning at home. ECEC is defined as arrangements for providing care and education for children under formal school age (compulsory school attendance in Australia is from age 6). EC educators are critical to lifting the quality of early learning programs and supporting the emotional and developmental outcomes of young children (Cassidy et al., 2017; Langeloo et al., 2019; Melhuish et al., 2015; Perlman et al., 2016; Shonkoff et al., 2012; Shuey & Kankaraš, 2018). Given this, identifying strategies to support and sustain EC educator wellbeing is particularly important as a response to the global pandemic and provides lessons for both usual practice and future critical contexts. Improving EC educators’ wellbeing is not only intrinsically important for the profession, but it will also help to ensure a sustainable and high-quality workforce, with benefits for children, families, and communities.

### What We Know about EC Educator Wellbeing

Studies examining EC educator wellbeing reflect a holistic concept of wellbeing, where the individual markers of physical, psychological, social, and emotional health are considered alongside systemic burdens, resources to support staff, and the culture of the workplace (Liu et al., 2018). In other words, elements of wellbeing are shaped by individual factors such as physical health and mechanisms for coping with stress, as well as external factors such as the work environment and social and political contexts (Benevene et al., 2018; Logan et al., 2020; OECD, 2019).

Characteristics of individual EC educators that have been found to support wellbeing and lower stress include resilience (the capacity to recover from difficult events or experiences), mindfulness (the practice of being fully present and engaged in the moment, aware of ones thoughts and feelings without distraction or judgement), self-compassion (acceptance of one’s own shortcomings and understanding that suffering and personal failure is part of the shared human experience), and self-efficacy (EC educator’s belief that they have the capability to support children’s learning and development) (Bouillet et al., 2014; Jennings, 2015). For example, a synthesis of teacher self-efficacy (TSE) research found that TSE was positively associated with students’ academic adjustment, teacher practices related to classroom quality, and teachers’ psychological wellbeing, while negative associations were found between TSE and burnout factors (whereby burnout is defined as psychological and/or physical exhaustion caused by prolonged, high levels of stress) (Zee & Koomen, 2016). A study in the USA assessing the effectiveness of a mindfulness program found that EC educators who practiced mindfulness had higher social and emotional competence, with positive impacts on the quality of their emotionally responsive interactions in the classroom (Jennings, 2015). In Hong Kong, Wong and Zhang (2014) studied 371 kindergarten (EC) educators' wellbeing, perceived school culture, and personality types, and found that EC educators with more extraverted personalities tended to perceive their school culture more positively and have higher levels of job satisfaction and self-esteem than those teachers who were more introverted.

Meanwhile, contextual factors that have been found to negatively influence EC educator wellbeing and retention include low wages, overwhelming workloads, unsupportive management, lack of autonomy, stressful encounters with colleagues and parents, and managing children’s challenging behaviors (Corr et al., 2015; Jena-Crottet, 2017; Jones et al., 2017; Li & Zhang, 2019; Logan et al., 2020). Professional status and feeling valued by society have a significant impact on EC educators’ sense of worth and wellbeing, and consequently their practice (Irvine et al., 2016; Logan et al., 2020; OECD, 2019). For example, the OECD’s Starting Strong Teaching and Learning International Survey (2018) found that while overall job satisfaction was high among EC professionals, staff reported feeling more valued by children than by society in general. Further, feeling valued by society was associated with greater use of practices that supported individual children’s development and interests. In Australia, findings from a national Early Childhood Education and Care Workforce survey (Irvine et al., 2016) indicated that while the majority of EC educators recognize the importance of their work with children, they experience high levels of stress and challenging working conditions which are exacerbated by working in a society that fails to value the professional quality of their work.

### The Importance of EC Educator Wellbeing for Relationships with Young Children and Their Learning

Given that EC educator wellbeing is inextricably linked to program quality, poor EC educator wellbeing has significant implications for children’s learning and development outcomes (Corr et al., 2015; Jena-Crottet, 2017; King et al., 2016; Logan et al., 2020; Smith & Lawrence, 2019). EC educators are the central enabling influence on the quality of program delivery, and high-quality programs can have significant short- and long-term benefits for all children, especially for children experiencing disadvantage (Campbell et al., 2014; Duncan & Magnuson, 2013; Goldfeld et al., 2016; Melhuish et al., 2015; Shonkoff et al., 2012). In particular, the importance of responsive and respectful EC educator-child relationships is well established in the literature (Burchinal et al., 2016; Pianta et al., 2016; Slot, 2018; Tayler et al., 2016; Torii et al., 2017) and has been identified as a key area of practice in the Australian National Quality Framework (NQF) (Quality Area 5: Relationships with Children; ACECQA, 2009). The NQF provides a national approach to assessment, regulation and quality improvement for a range of ECEC services including family day care, long day care, preschool/kindergarten (defined as an early childhood education program in the year prior to formal schooling) and outside school hours care across Australia. The NQF introduced a suite of documents which align and embed clear expectations and accountabilities to ensure the continuous improvement of quality teacher and improved learning outcomes for young children attending ECEC services (Page & Eadie, 2019). There is consensus that high quality EC educator-child interactions have the greatest impact on ECEC program quality and, importantly, on children’s learning and development outcomes (OECD, 2019; Slot, 2018; Torii et al., 2017). However, the provision of sustained and responsive interactions with children is predicated on EC educators’ wellbeing and their emotional and mental capacity to excel in their role (Jennings, 2015; Kim & Choi, 2018; Roberts et al., 2016).

Behaviors that predict wellbeing, such as mindfulness and self-efficacy, are associated with higher quality EC educator-child interactions, especially in relation to sensitivity and behavioral support (Jennings, 2015; Jennings et al., 2017). EC educators who are emotionally and mentally well are better equipped to be responsive to every child, to develop respectful EC educator-child relationships, and to support children’s confidence and engagement in learning (Buettner et al., 2016; Cassidy et al., 2017; Castle et al. 2016; Jennings, 2015; Pakarinen et al. 2010; Roberts et al. 2016). Furthermore, studies show that low levels of EC educator stress are associated with higher classroom organization and higher levels of engagement and motivation in children (Pakarinen et al., 2010).

Conversely, several correlational studies have shown that EC educators with high levels of depression and stress, emotional exhaustion or burn-out, are less likely to engage in high-quality interactions or emotionally responsive teaching practices (Buettner et al., 2016; Cassidy, et al., 2017; Jeon et al., 2014; Kim & Choi, 2018; Roberts et al. 2016). High levels of work-related stress are also associated with a reduction in professional commitment (Buettner et al. 2016) and higher rates of turnover (Phillips et al., 2016; Totenhagen et al. 2016). High rates of EC educator turnover represent both the loss of EC educator skill and experience in the sector and a disruption to EC educator-child relationships, with significant adverse implications for children’s learning and social-emotional outcomes (Cassidy et al. 2017; National Scientific Council on the Developing Child 2015). Of particular concern is that turnover rates are higher in early childhood centers under greatest stress, including centers attended by children living in disadvantaged circumstances (Allen et al., 2018; Stormont & Young-Walker, 2017; Wells, 2017).

As poor wellbeing of EC educators can disrupt relationships with children and threaten the quality of EC educators’ practice, identifying strategies that can support and sustain healthy EC educator wellbeing is crucial to making quality improvements in the ECEC sector. Research has identified individual and environmental factors that influence teacher/EC educator wellbeing, but studies have not yet accounted for the additional burden of remote learning, supporting families experiencing hardship, and the pervasive personal and professional demands of rapidly changing impacts of the COVID-19 pandemic. In this study, we aimed to explore EC educators’ wellbeing during the COVID-19 pandemic, and the protective and risk factors associated with EC educator wellbeing and responsive EC educator-child relationships during the pandemic and beyond. The specific research questions, within the context of the COVID-19 pandemic, were:

- How do EC educators rate the impact of COVID-19 on their wellbeing and their relationships with children?
- What are the protective and risk factors (e.g., years of experience, type of EC service) for the professional wellbeing of EC educators?
- To what extent does EC educators’ professional wellbeing impact on the risk of staff turnover?
- To what extent does EC educators’ professional wellbeing impact on the nature of educator-child relationships?

## Methods

### Participants

A cross-sectional survey design was employed to investigate the research questions. EC educators were recruited through a Research Network of Early Childhood Professionals and via our broader ECEC networks between July and October 2020 via a link to an online survey. Prior to completing the survey, participants were provided with information about the study and an online consent form. Participation was voluntary and participants were informed that their information would be confidential and securely stored. All participants provided informed consent. Ethical approval for the study was obtained from the University of Melbourne Human Research Ethics Committee (#1954943).

The survey took participants approximately 20 min to complete: 232 participants completed the ECPW scale, and 215 completed the Student–Teacher Relationship Scale (STRS). The 232 respondents who provided complete ECPW data are included in the analyses that follow.

### Measures

An EC educator survey was used to identify elements of wellbeing central to EC educator efficacy, EC educator-child relationships, and the risk of staff turnover. The method of an online survey was best suited to the restrictions of social contact imposed by COVID-19, to seek input from EC educators across Australia, and to collect data with minimal intrusion for participants given the additional burdens already experienced by EC educators. The survey was comprised of three components: (1) demographic questions designed by the research team (e.g., role, years of experience, working hours, service type, location, age of children); (2) study-designed questions about the extent to which EC educators felt that the COVID-19 pandemic had impacted on their wellbeing and their relationships with children; (3) the Early Childhood Professional Wellbeing (ECPW) scale (McMullen, et al., 2020); and (4) the Student–Teacher Relationship Scale (STRS) short form (Pianta, 2001) modified for staff wellness (see Whitaker et al., 2015).

The study-designed questions relating to the impact of the pandemic on wellbeing and EC educators’ relationships with children asked EC educators to indicate on a five-point Likert scale (where 1 = Strong negative impact and 5 = Strong positive impact) to what extent the COVID-19 pandemic impacted on: (a) Your wellbeing; and, (b) Relationships with children.

The ECPW scale was designed to measure EC educators’ wellbeing and risk of turnover. The framework is underpinned by sociocultural theory, with wellbeing based on nine ‘senses’ that capture Maslow’s hierarchy of needs: Agency, Efficacy, Engagement, Contribution, Affinity, Self-Respect, Communication, Security, and Comfort. Exploratory factor analyses (McMullen et al., 2020) determined a three-factor model which retained all 27 individual items best aligned with the theoretical constructs underpinning the ECPW scale: that is, factors that represented Collegial Relationships, Supportive Structures, and Professional Beliefs and Values. Eleven items from across all nine senses are included in the Supportive Structures factor, seven items from four senses are included in the Collegial Relationships factor and nine items from five senses are included in the Professional Beliefs and Values factor. An overall score for the ECPW scale is calculated by adding the score (on a five-point Likert scale) for each of the 27 wellbeing items, such that an overall score can range from 27 to 135 with higher scores indicating higher professional wellbeing. Risk of turnover is calculated as the total of the three turnover items (on a five-point Likert scale), ranging from 3 to a maximum of 15, with higher scores indicating less risk of turnover.

The STRS short form is one of the most widely used instruments to assess the quality of the emotional relationship between a teacher and a child and has been shown to have high discriminant validity (Hamre & Pianta, 2001; Pianta, 2001). The STRS assesses the teacher’s perception of their relationship with an individual child and produces two subscale scores: closeness and conflict. The modified STRS was adapted by Whitaker et al. (2015) in consultation with the developer of the STRS, Robert Pianta. Modifications to the original 15 items were made so that respondents are asked about the emotional climate of the classroom rather than asking about an individual child. The modified STRS asks 15 items identifying child and teacher behaviors that reflect the quality of relationships in the classroom in general (e.g., “If upset, the children will seek comfort from me”). As with the STRS short form, the modified STRS asks teachers to rate on a 5-point Likert scale (where 1 = definitely does not apply, and 5 = definitely applies) the degree to which the items apply to their relationship with the children in their classroom. The 15 items of the modified STRS produce two subscale scores: closeness and conflict. The conflict subscale has eight items to capture negative, insecure, and hostile aspects of relationships, while the closeness subscale has seven items to assess warmth, security, and openness in relationships. Subscale scores are calculated by summing items for each subscale. The range of possible scores are: 8–40 for conflict and 7–35 for closeness. Higher scores indicate a higher level of conflict and closeness.

### Analyses

Descriptive statistics were calculated for EC educator wellbeing (ECPW Scale and study-generated item), EC educator-child relationship (STRS and study generated item), and participant characteristic variables. Bivariate statistics, examining the relationship between two variables, were used to examine the associations between participant characteristics (potential risk and protective factors including participant role, service location, service type, age of children with whom participants were working, and years of experience) and EC professional wellbeing. The state (in Australia) was included as a potential risk factor in the bivariate analysis as the state of Victoria, unlike other parts of Australia, was in lockdown for several months during the data collection period: ECEC services in metropolitan Melbourne were closed to families for a period of 8 weeks, with the exemption of vulnerable children and children of essential workers. Therefore, the state where EC educators were living and working could potentially be associated with professional wellbeing within the COVID-19 context.

To examine the extent to which professional wellbeing was associated with risk of turnover during the pandemic, correlations were calculated for overall wellbeing, the three factors of wellbeing and risk of turnover.

Linear regression (analysis that provides estimates to explain the relationship between one dependent variable and one or more independent variables) was then used to examine the relationship between the EC educator-child relationship subscales of closeness and conflict and professional wellbeing. Professional wellbeing was included as a predictor variable (continuous) in separate unadjusted (crude) regression analyses to determine if professional wellbeing predicted closeness and conflict, and this was further extended to adjusted analyses, to examine if professional wellbeing was associated with closeness and conflict above the potential confounders (participant characteristics from bivariate analyses that had a p-value of 0.1 or smaller were included). All analyses were conducted using the integrated statistical software Stata16 (StataCorp, 2019).

## Results

### EC Educator Wellbeing and Relationships with Children

Of the 353 respondents who consented to take part in the survey, 232 participants provided wellbeing data, while 215 provided EC educator-child relationship data. Table 1 shows participant characteristics. Participants’ roles included Lead teacher/Educator (35.5%), Assistant teacher/ Educator (22.5%), Educational Leader (16.0%), Center Director (13.4%) and Family Day Care Educator/Owner (7.4%). Almost two-thirds of participants were from Victoria (70.6%) and 72.5% were working in metropolitan areas across Australia. Participants’ experience was measured in 5-year intervals, and the largest proportion of participants reported 5–10 years of experience (22.9%) and the smallest proportion reported 15–20 years (9.5%). Almost a quarter of all participants had 25 or more years of experience. A large proportion of participants reported working with 3–5-year-olds (46.2%) and mixed ages of children (39.1%). Just over half of the participants reported working at center-based long day care (private: 23.0%; non-profit: 29.2%), while around a third reported working at a kindergarten/preschool (private: 8.4%; non-profit: 25.2%).

**Table 1:** Descriptive statistics and bivariate associations between participant characteristics and educator professional wellbeing

Variable	*n* (%)	Bivariate association β [95%CI], *p*
Participant role (n = 231)		
Assistant teacher/Educator	52 (22.51)	Reference
Lead teacher/Educator	82 (35.50)	0.96 [− 3.78, 5.70], 0.69
Educational leader	37 (16.02)	0.80 [− 4.94, 6.55], 0.78
Center director	31 (13.42)	7.33 [1.27, 13.39], 0.018
FDC educator/owner	17 (7.36)	8.32 [0.86, 15.79], 0.029
Other	12 (5.19)	8.22 [− 0.33, 16.78], 0.059
Years of experience (n = 231)		
Less than 5 years	34 (14.72)	Reference
5–10 years	53 (22.94)	1.33 [− 4.55, 7.21], 0.66
10–15 years	38 (16.45)	4.07 [0.21, 10.38], 0.21
15–20 years	22 (9.52)	1.15 [− 6.17, 8.47], 0.76
20–25 years	27 (11.69)	7.06 [0.16, 13.95], 0.05
25–30 years	29 (12.55)	3.68 [− 3.08, 10.44], 0.28
More than 30 years	28 (12.12)	9.83 [3.00, 16.65], 0.005
Age of children (n = 225)		
Birth-2 years	14 (6.22)	Reference
2–3-year olds	19 (8.44)	1.24 [− 8.19, 10.68], 0.80
3–5 year olds	104 (46.22)	5.47 [− 2.16, 13.09], 0.16
Mixed ages	88 (39.11)	8.20 [0.50, 15.91], 0.037
Service type (n = 226)		
Center-based LDC (private)	52 (23.01)	Reference
Center-based LDC (non-profit)	66 (29.20)	0.18 [− 4.79, 5.15], 0.94
Kindergarten/preschool (private)	19 (8.41)	3.68 [− 3.50, 10.87], 0.31
Kindergarten/preschool (non-profit)	57 (25.22)	1.63 [− 3.51, 6.77], 0.53
Family day care	22 (9.73)	10.4 [1.14, 19.66], 0.028
Occasional care	10 (4.42)	6.32 [− 0.50, 13.14], 0.069
Service location (n = 229)		
Metropolitan	166 (72.49)	Reference
Regional	44 (19.21)	− 4.67 [− 5.11, 4.17], 0.84
Rural	17 (7.42)	− 1.63 [− 8.60, 5.34], 0.47
Remote	2 (0.87)	− 7.22 [− 26.68, 12.24], 0.47
State (n = 228)		
Victoria	161 (70.61)	Reference
New South Wales	39 (17.11)	0.81 [− 4.09, 5.71], 0.75
South Australia	4 (1.75)	− 5.38 [− 19.28, 8.52], 0.75
Western Australia	7 (3.07)	1.52 [− 9.09, 12.12], 0.78
Tasmania	2 (0.88)	8.37 [− 11.17, 27.91], 0.58
Queensland	15 (6.58)	2.11 [− 5.31, 9.52], 0.58

Regarding the extent to which EC educators felt that the pandemic had impacted on their wellbeing, 85.9% (183/213) reported a strong negative or slight negative impact. When reporting on their relationships with children, 37.1% (79/213) indicated the pandemic had a strong negative or slight negative impact, while 23.5% (50/213) reported a slight positive or strong positive impact.

Table 2 shows the descriptive statistics for professional wellbeing, as measured using the ECPW scale and EC educator-child relationships, measured using the STRS. Means and standard deviations are presented for the average overall professional wellbeing score, the nine senses scores, factor scores and risk of turnover score. Means and standard deviations are also present for the two dimensions of the STRS, closeness and conflict. Of the three factors of wellbeing, average scores for Supportive Structures (environment and climate: M = 39.5; SD = 6.2) and Professional Beliefs & Wellbeing (autonomy, choices, participation in decision making: M = 37.3, SD = 4.8) were higher than Collegial Relationships (between/among adults: M = 27.1, SD = 4.6). Of the nine senses of wellbeing, sense of contribution had the highest score (M = 12.26, SD = 2.04) and communication with other adults in the workplace had the lowest average score (M = 10.9, SD = 2.0). Regarding the EC educator-child relationships during the pandemic, results showed that the average closeness score was high (M = 36.7, SD = 4.1) and conflict score was low (M = 13.50, SD = 4.3).

**Table 2:** Educator professional wellbeing and educator-child relationship descriptives

Variable	M (SD)	Min, Max
Early childhood professional wellbeing scale (n = 232)	103.97 (13.76)	64, 131
Supportive structures	39.53 (6.21)	20, 52
Collegial relationships	27.14 (4.61)	13, 35
Professional beliefs & values	37.30 (4.84)	19, 45
Risk of turnover	10.28 (2.70)	4, 15
Senses of wellbeing		
Comfort	11.41 (2.10)	4, 15
Affinity	11.58 (2.25)	5, 15
Self-respect	12.06 (2.27)	5, 15
Communication	10.93 (2.01)	4, 15
Engagement	11.88 (1.81)	6, 15
Contribution	12.26 (2.04)	6, 15
Efficacy	11.25 (1.53)	7, 15
Agency	11.22 (2.09)	4, 15
Security	11.39 (2.10)	5, 15
Educator-Child Relationship Scale (n = 215)		
Closeness	36.65 (4.08)	17, 40
Conflict	13.50 (4.34)	7, 31

### Associations Between Participant Characteristics and Professional Wellbeing

There was evidence of a bivariate association between professional wellbeing and participants role within EC services (see Table 1). Table 1 shows that professional wellbeing scores were significantly higher for those in Center Director (β = 7.33, 95% CI: 1.27–13.39, *p* = 0.018) or Family Day Care Educator/Owner (β = 8.32, 95% CI: 0.86 to 15.79, *p* = 0.029) roles compared to Assistant Teacher/ Educator roles during the pandemic. Participants with more than 30 years of experience had average professional wellbeing scores significantly higher than those with less than 5 years of experience (β = 9.83, 95% CI: 3.00 to 16.65, *p* = 0.005). The overarching association between years of experience and professional wellbeing approached (*p* = 0.06) but was not statistically significant. EC educators working with mixed age groups reported, on average, higher wellbeing scores compared to those EC educators working with birth to 2-year-olds (β = 8.20, 95% CI: 0.50 to 15.91, *p* = 0.037) (see Table 1). The association between the age of children that EC educators worked with and their wellbeing similarly approached (*p* = 0.06) but was not statistically significant.

### Associations Between Professional Wellbeing and Risk of Turnover

As per McMullen et al (2020), correlations were calculated to look at the association between risk of turnover and the three factors of wellbeing, as well as overall professional wellbeing. Stronger professional wellbeing was found to be associated with lower risk of turnover (*r*(232) = 0.63, *p* < 0.001). Of the three wellbeing factors, Supportive Structures was most strongly associated with lower risk of turnover (*r*(232) = 0.63, *p* < 0.001), and there was also evidence of associations between Professional Values & Beliefs (*r*(232) = 0.47, *p* < 0.001) and Collegial Relationships (*r*(232) = 0.55, *p* < 0.001) with a lower risk of turnover.

### Associations Between Professional Wellbeing and EC Educators’ Relationships with Children

Unadjusted and adjusted regression models were used to examine the association between professional wellbeing and teacher–child relationships (Table 3). In unadjusted regression models, higher professional wellbeing was negatively associated with higher conflict scores, indicating that in this sample, stronger professional wellbeing was associated with less conflict in EC educators’ relationships with children. Professional wellbeing explained 13.8% of the variation in conflict scores. There was no evidence of an association between professional wellbeing and scores on the closeness scale.

**Table 3:** Unadjusted and adjusted associations between professional wellbeing and Educator-Child Relationship Scale

	Unadjusted β [95% CI], *p*	R^2^ (%)	Adjusted^a^ β [95% CI], *p*	R^2^ (%)
STRS conflict		13.75		18.57
ECPW	− 0.11 [− 0.15, − 0.07], < 0.001		− 0.10 [− 0.15, − 0.06], < 0.001	
STRS Closeness		1.68		9.67
ECPW	0.04 [− 0.002, 0.07], 0.061		0.03 [− 0.01, 0.07], 0.19	

^a^Adjusted for participant role, years of experience and age of children

Fully adjusted models were used to account for participant characteristics that were shown to be associated with professional wellbeing in the bivariate analyses (i.e., participant role, years of experience, and age of children). The association between professional wellbeing and conflict in relationships with children remained after adjusting for participant characteristics (i.e., potential confounding variables). The fully adjusted model explained 18.6% of the variation in conflict scores. There was no evidence of an association between any of the potential confounders added to the model with conflict as the outcome. As with the unadjusted results, there was no evidence of an association between wellbeing and closeness in fully adjusted models.

## Discussion and Implications

COVID-19 brought attention to the essential work of the early childhood profession in ways that ‘normal’ circumstances had not been able to achieve over many years, highlighting vulnerabilities in the early childhood sector largely overlooked by policy makers and governments. The results of our survey conducted during the height of significant disruptions to workplaces in Australia provided insights into the lived experience of early childhood professionals. When asked to rate the impact of COVID-19 on their wellbeing, (research question 1), the majority of EC educators reported a negative impact. However, on the ECPW Scale, the average scores for professional wellbeing were in the higher range, reflected in all three of the measure’s factors: highest scores for Professional Beliefs and Values, followed by Collegial Relationships, and then Supportive Structures. The juxtaposition of outcomes between EC educators’ perception of wellbeing and their scores on the ECPW may be partially explained by the supports that had been put in place at a policy level (e.g., financial) and at a service level (e.g., wellbeing supports and working conditions). While the broader societal conditions, particularly in metropolitan Victoria, which was in the strictest lockdown, may have been related to EC educators’ global ratings of negative impact, the EC sector was responding flexibly and providing some supports for the workforce, potentially bolstering EC educator wellbeing. In our sample of EC educators, we found they reported strong relationships with young children, reflected in higher closeness scale scores than those in the Whitaker et al. (2015) and lower conflict scores.

In relation to the impact of the pandemic, significantly fewer EC educators reported a negative impact on their relationships with children (37%) compared to the negative impact on their wellbeing (86%), with nearly a quarter of EC educators indicating a positive impact on their relationships with children. This suggests that EC educators felt they were able to sustain strong relationships with children despite the perceived negative impact of the pandemic on their wellbeing. Further, on the ECPW, EC educators rated their sense of contribution highest. This correlates with previous workforce studies showing that EC educators value and recognize the intrinsic importance of their work with children despite external challenges (Irvine et al., 2016; OECD, 2019) and may also reflect the spotlight that was shone on the ECEC sector during the pandemic, acknowledging ECEC as an essential service. In addition, the fact that EC educators rated their sense of communication with other adults lowest in the ECPW does not seem surprising given the restricting conditions necessitated by COVID-19, reducing opportunities for collegial ‘corridor’ conversations in the workplace.

Participants with more senior roles in services, longer work experience in the sector, and who worked with mixed age groups of children (rather than children under 2 years) reported stronger wellbeing on the ECPW (research question 2). Associations between years of work experience and a senior work role, and EC educator wellbeing, has been linked in other research to lower risk of turnover (Thorpe et al., 2020).

Higher overall wellbeing scores and higher scores on the Supportive Structures factor were associated with lower risk of turnover in our participant group (research question 3). While higher wellbeing scores on all three factors were associated with lower risk of staff turnover, our findings replicate previous research by McMullen et al., (2020) where Supportive Structures was the most important factor related to turnover. These findings suggest that even in exceptional circumstances, such as a global pandemic, what supports EC educators’ wellbeing remains relatively stable. Supportive organizational structures and culture can assist EC educators to meet the demands and expectations of their role, develop quality practice, ensure supportive working environments, and increase an EC educator’s intention to stay (Irvine et al., 2016; Logan et al., 2020).

Finally, we investigated the association between EC educators’ wellbeing and their relationships with children (research question 4). Stronger EC educator wellbeing was associated with less conflict, and this association was still evident after adjusting for potential confounding variables. Conversely, no association was found for the closeness scale on the STRS. The findings in the present study support previous research where wellbeing has been found to correlate with EC educators’ use of emotionally responsive and high-quality interactions (Buettner et al., 2016; Cassidy et al., 2017). We hypothesize that conflict is often perceived during in-the-moment interactions where stronger wellbeing can mitigate the likelihood of conflict occurring in the first place. On the other hand, EC educators’ perceptions of closeness are more likely related to the overall climate and relationships in a room, rather than specific interactions. This provides a potential explanation for a lack of an association between closeness and wellbeing ratings, during a year when children and EC educators were apart for extended periods of time.

### Limitations and Future Research

It is important to acknowledge the limitations of this study. The sample was self-selected and over two-thirds of participants were from Victoria. In addition, as data collection was via an online survey, only those with internet access were able to participate. Therefore, respondents may not be representative of the broader population of EC educators in Australia. However, the over-representation of participants from Victoria provides useful insights into the impact of the pandemic on EC educator wellbeing and EC educator-child relationships given this state—in particular, metropolitan Victoria—suffered the most extreme lockdown, both regarding severity of restrictions and duration of lockdown. While a self-report survey design is an efficient method for collecting data from a large sample to address the research questions, a threat to the validity of self-report is socially desirable responding. To address this, respondents were assured that their answers were anonymous and confidential. Future research including qualitative methods, taking a mixed methods approach, would assist to understand any contradictions between quantitative results and qualitative findings, by gathering in-depth information from participants. Focus groups were conducted with a sub-sample of EC educators from the current study and the aim was to analyze and present this data to compliment the current findings. It must also be noted that this study was cross-sectional, so causal pathways cannot be examined. It is recommended that future longitudinal research be conducted to determine possible bidirectional relationships between EC educator wellbeing and EC educator-child relationships, as well as risk of staff turnover. Future research should also aim to examine the effectiveness of interventions for improving educators’ wellbeing and preventing work-related stress, using high-quality randomized control trials.

### Conclusions

The fundamental importance of supporting EC educator wellbeing is increasingly recognized as a professional responsibility for everyone in the field of ECEC (Cumming & Wong, 2018). This has been further highlighted through the context of COVID-19. The global pandemic has resulted in increased demands on EC educators. Rather than assuming these demands directly affected EC educator wellbeing, our study aligns with previous research to show that the stressors of a traumatic event, such as the pandemic, have exposed *existing* cracks in the system and supports for early childhood education. This study confirms the need for leaders and managers in ECEC services to focus on EC educators’ wellbeing and the factors that support it. The effectiveness of leaders in this regard is most likely to impact on EC educators’ wellbeing and, in turn, support retention of EC educators’ skills and experience in the sector. There are significant lessons to be learned from COVID-19 regarding the resilience of the workforce, the visibility (or not) of the important work of EC educators, the value society places on the work, the level of government investment in supporting EC educators in the sector and the role that wellbeing plays in this (McMullen et al., 2020). These lessons are vital to ensuring EC educators, in turn, can be supported and acknowledged for the critical role they play in advancing young children’s social, emotional and cognitive learning, wellbeing, and development.
